# Development of a Potential Bone-Seeking Radiopharmaceutical by Sodium Pyrophosphate Labeled ^188^Rhenium (^188^Re-PYP) for Bone Pain Palliation

**DOI:** 10.5812/ijpr-153691

**Published:** 2025-03-09

**Authors:** Mahmoud Moradi, Mehdi Salehi Barough, Leila Moghaddam-Banaem, Fariba Johari Daha, Sahar Rajabi-Moghadam

**Affiliations:** 1 Department of Medical Radiation Engineering, Central Tehran Branch, Islamic Azad University, Tehran, Iran; 2 Nuclear Science and Technology Research Institute (NSTRI), Tehran, Iran; 3 Radiation Application School, Nuclear Science and Technology Research Institute (NSTRI), Tehran, Iran

**Keywords:** Rhenium-^188^, Pyrophosphate, Radio-Labeling, Biodistribution

## Abstract

**Background:**

Technetium-^99m^ (^99m^Tc)-pyrophosphate (PYP) has been widely utilized in diagnosing bone disorders and certain cardiac conditions, such as amyloidosis, allowing for accurate imaging and detection of abnormalities within heart tissue. Rhenium, being in the same group as technetium in the periodic table, shares similar chemical properties. Rhenium-^188 ^(^188^Re) possesses favorable nuclear properties for theranostic applications.

**Objectives:**

This study focused on labeling sodium PYP with ^188^Re and its biodistribution.

**Methods:**

Different samples with varying amounts of PYP (5 - 22 mg), SnCl_2_.2H_2_O (0.2 - 6.0 mg), and ascorbic acid (0.3 - 7 mg) were prepared in vials. Initially, 0.08 mg of potassium perrhenate as a carrier in 1 mL saline was added to each vial. Subsequently, 370 - 3700 MBq of ^188^ReO_4_^−^ was added to the initial solution. The pH of the solutions was varied between 3 and 8. The compound was shaken vigorously for 30 seconds. Product incubation was performed in a secured container for 30 minutes at room temperature.

**Results:**

Maximum labeling yield was achieved with 10 mg of PYP, 1 mg of SnCl_2_.2H_2_O, 0.3 mg of ascorbic acid, and 0.08 mg of potassium perrhenate as a carrier in 1 mL with 370 MBq of ^188^ReO_4_^−^ at pH 5. This compound showed good stability, and a radiochemical purity of 98.96% ± 0.1% was obtained. The biodistribution results of the radiolabeled ligand revealed that the maximum affinity for ^188^Re-PYP was for bone after 4 hours, which was 2.24% ± 0.667% ID/g. The maximum uptake for the kidney, spleen, and liver was 1.53% ± 0.378%, 0.13% ± 0.086%, and 0.18% ± 0.12% ID/g, respectively.

**Conclusions:**

The present study investigated the initial labeling efficiency of ^188^Re-PYP along with its biodistribution and in vitro stability. The ^188^Re-PYP conjugate, prepared under optimized conditions, demonstrated radiochemical purity and stability. The biodistribution of the compound in mice exhibited high affinity for bone, whereas the complex was eliminated through the kidneys.

## 1. Background

Radiopharmaceuticals play a vital role in nuclear medicine, enabling physicians to diagnose and treat various medical conditions through imaging and targeting techniques that utilize radioisotopes. One important radiopharmaceutical is ^99m^ technetium-pyrophosphate (^99m^Tc-PYP), which holds significant value in nuclear medicine due to its bone-seeking properties. It has been widely used for the diagnostic imaging of bone diseases and certain cardiac conditions, such as amyloidosis (1-4). Hydroxyapatite and calcium phosphates in bone have a tendency to bind bisphosphonates, making these molecules preferred for bone scans. Most bisphosphonates have a hydroxyl group in a carbon position that binds to calcium phosphate with great affinity, and they also possess a highly reducing group with anti-adsorbing power in another carbon position (5-9). These properties make bisphosphonates preferred for diagnostic and therapeutic applications in nuclear medicine.

The first-generation bisphosphonates, such as etidronate, contain short side chains. The second-generation compounds, alendronate and olpadronate, contain aliphatic chains of varying lengths that include amino mass terminals. Third-generation bisphosphonates, such as risedronate and zoledronic acid, possess heterocyclic nitrogen side chains that are important for hydroxyapatite crystals in bone, and these agents are anti-resorptive. This anti-resorptive characteristic makes third-generation bisphosphonates at least 100 to 1000 times more potent than etidronate and pamidronate. At the cellular level, risedronate decreases bone turnover and inhibits osteoclasts (10-12).

Pyrophosphate (PYP), a molecule with an affinity for calcium ions found in bones, acts as a "bone seeker" when combined with radionuclides. Pyrophosphate is a phosphorus oxyanion composed of two phosphate units linked by a P-O-P bridge, with the molecular formula P_2_O_7_^4-^. Pyrophosphate forms complexes with various metals, which is critical for its use in radiopharmaceuticals. It is highly soluble in water and exhibits a strong affinity for calcium ions, making it effective in targeting bone tissue where calcium deposition occurs (13-15). The absorption of four polar components by lanthanum-loaded biochar (BC-La), including phytic acid (IHP), adenosine-5′-disodium triphosphate (5-ATP), hydroxyethylidene diphosphonic acid (HEDP), and sodium PYP, was investigated by Yuan et al. (16). The results revealed that the maximum adsorption of BC-La for IHP, 5-ATP, HEDP, and PYP was 85.85, 9.04, 15.80, and 14.45 mg/g, respectively. Although the absorption of PYP is lower than that of bisphosphonates, the advantages of easier labeling with PYP and greater stability of radiopharmaceuticals with PYP-labeled agents make PYP an alternative to other bone-seeker ligands. An investigation (17) showed that labeling PYP with ^177^Lu achieved a maximum yield (> 99%) with only one minute of incubation at room temperature.

Emitters with low-energy β^−^ are radionuclides such as ^153^Sm, ^177^Lu, ^186^Re, and ^175^Yb, which are applied for bone pain alleviation, while high-energy radionuclides such as 188Re, ^90^Y, and ^166^Ho are beneficial in marrow ablation due to bone composition (5-7, 10-12, 18-20). Another useful radionuclide is technetium-^99m ^(^99m^Tc). Technetium is a transition metal with the atomic number 43 and is primarily used in its isotope form, Tc-^99m^, in medical applications. Tc-^99m^, with a 6-hour half-life and gamma photon emission at 140 keV energy, is ideal for medical imaging. Technetium forms various chemical compounds and can exist in multiple oxidation states, with Tc (VII) and Tc (IV) being the most common in radiopharmaceutical chemistry. The chemistry of technetium is complex, allowing it to form stable complexes with ligands like PYP, enhancing its imaging capabilities (21).

Historically, ^99m^Tc labeled PYP (Tc-PYP) has been widely utilized due to its excellent imaging properties, making it a cornerstone in diagnosing bone disorders and certain cardiac conditions, such as amyloidosis. This allows for accurate imaging and detection of abnormalities within heart tissue. The mechanism of localization involves an influx of calcium after cell death in acute myocardial infarction, leading to the formation of calcium phosphate complexes. These microcrystalline deposits act as sites for ^99m^Tc-PYP uptake. When introduced into the body, PYP radiopharmaceutical accumulates in areas of increased bone metabolism or damaged bone tissue, making it ideal for identifying bone diseases and injuries such as fractures, tumors, infections, and cancer metastasis (15).

Rhenium is a transition metal with the atomic number 75, and its isotope, rhenium-^188^ (^188^Re), is of particular interest in therapeutic applications. Rhenium is in the same group as technetium in the periodic table, giving it chemical properties similar to technetium. Rhenium-^188^ has a half-life of 16.9 hours and emits high-energy beta particles (2.12 MeV maximum energy), effective for targeted radionuclide therapy. It also emits gamma photons (155 keV), applicable for imaging. Like technetium, ^188^Re exhibits multiple oxidation states, with Re (VII) and Re (V) being the most relevant for radiopharmaceuticals. The chemical properties of rhenium allow it to form stable complexes with ligands like bisphosphonates, ensuring effective delivery to target tissues while sparing surrounding healthy cells (22).

In this study, labeling PYP with ^188^Re is investigated. The combination of PYP labeled with ^188^Re enhances the capabilities of bone-seeker radiopharmaceuticals. Labeling PYP with ^188^Re involves attaching the radioisotope to the PYP molecule through specific chemical reactions. The labeling process is critical as it ensures stable binding between ^188^Re and PYP, allowing for effective radiation delivery to targeted sites within the bones. This study aims to explore methodologies for efficient labeling, evaluate the in vitro and in vivo stability of the ^188^Re- PYP complex, and assess its biodistribution and therapeutic efficacy in relevant animal models.

## 2. Objectives

By advancing the knowledge of ^188^Re-labeled compounds, this research contributes to developing novel therapeutic strategies for bone-related diseases, including metastatic bone cancer and osteosarcoma. Integrating diagnostic and therapeutic capabilities in a single agent presents a transformative approach in personalized medicine, offering targeted treatment while concurrently monitoring therapeutic outcomes. These dual capabilities could streamline patient management, enhance treatment efficacy, and improve predictive accuracy.

## 3. Methods

### 3.1. Materials

The necessary chemicals and PYP were obtained from Sigma-Aldrich. The compound underwent natural rhenium analysis to obtain UV spectra, which were recorded using a Varian Cary3 spectrometer. The Tungsten-^188^/^188^Re generator from PARS-Isotope Company of Iran was used as the source of ^188^Re. A saline solution (0.9% NaCl) was used to extract ^188^ReO_4_^−^ from the alumina-based ^188^Re generator. A dose calibrator (Isomed, Germany) was used to measure the activity of ^188^ReO_4_^−^, which was 500 - 600 mCi. The radiochromatography analysis was conducted using Silica Gel ITLC chromatography paper from Agilent Technologies, US.

The activity distribution in the organs of mice was determined using the dose calibrator ISOMED 1010 (Dresden, Germany). The results were calculated as mean ± SD, and statistical analysis was performed using Student’s *t*-test, with P-values < 0.05 considered statistically significant. Male mice, with an average age of 8 ± 1 weeks, were sourced from the Nuclear Science and Research Institute (NSTRI) animal house. Animal studies adhered to the United Kingdom Biological Council’s guidelines.

All methods for preparation and quality control tests used in this investigation comply with the IAEA protocol for radiopharmaceutical production (23).

### 3.2. Production Along with Quality Control Assessment of the Rhenium-188-Pyrophosphate Compound

The experiment was conducted in two stages. The first stage involved the preparation of the compound in the cold phase using potassium perrhenate as a substitute for ^188^Re to obtain UV spectra. The second stage utilized ^188^Re to determine performance parameters, radiopharmacy processes, and biodistribution.

#### 3.2.1. Cold Phase of Experiment

A solution of PYP with a concentration of 30 mg/mL was prepared by dissolving PYP in water. Different samples with varying amounts of PYP (5 - 22 mg), SnCl_2_.2H_2_O (0.2 - 6.0 mg), and ascorbic acid (0.3 - 7 mg) were prepared in vials. In the first stage, 0.08 mg of potassium perrhenate as a carrier in 1 mL of saline was added to each vial. The final solutions from the first stage were utilized to obtain the UV spectra of the compound for assessing its chemical properties.

#### 3.2.2. Hot Phase of Experiment

In the second stage, 370 - 3700 MBq of ^188^ReO_4_^−^ was added to the solution from the first stage. The pH of the solutions was varied between 3 and 8. The compound was shaken vigorously for 30 seconds. Product incubation was performed in a secured container for 30 minutes at room temperature. The solutions from the second stage were used to check radiochemical purity by the ITLC method and to assess biodistribution.

### 3.3. Radiochemical purity of Rhenium-188-Pyrophosphate

Samples with micropipette volumes (5 μL) were taken from the ^188^ReO_4_^−^ solution and the radiolabeled complex, then dotted onto chromatography paper. Two solvent systems were utilized for the mobile phase in ITLC to discriminate the radiolabeled ligand from free ^188^ReO_4_^−^ and ^188^Re_2_O_4_: (A) Saline solution (0.9% NaCl) and (B) acetone. The ratio of free ^188^ReO_4_^−^ and ^188^Re_2_O_4_ to the radiolabeled ^188^Re-PYP was estimated.

In the saline solution, ^188^Re_2_O_4_ remained at the spot where it was applied, while the free ^188^ReO_4_^−^ and the compound moved with the solvent front, allowing for the determination of the percentage of free ^188^Re_2_O_4_. In the acetone solution, the complex and ^188^Re_2_O_4_ remained at the spot, while the free ^188^ReO_4_^−^ moved to the front, allowing for the determination of the percentage of free ^188^ReO_4_^−^. The percent radiochemical purity was obtained using Formula 1:

%Radiochemical purity = 100 - % ^188^Re_2_O_4_ - %^188^ReO_4_^−^

### 3.4. Investigation of In Vitro Stability of Rhenium-188-Pyrophosphate Compound

To study the compound's stability, it was incubated in human serum at 37°C and at room temperature for up to 72 hours and analyzed using ITLC. Rhenium-^188^-pyrophosphate (3.7 MBq in 100 μL) was added to 900 μL of freshly prepared human serum and stored at 37°C. At different time points (4, 24, and 48 hours after the reaction), 100 μL aliquots were removed and treated with 100 μL of ethanol (23). To precipitate the serum proteins, the samples were centrifuged at 3000 rpm for 10 minutes. Thereafter, chromatography of the supernatants was carried out.

### 3.5. Biodistribution of Rhenium-188-Pyrophosphate in BALB/c Mice

To assess the stability of the compound, it was incubated in human serum at 37°C and at room temperature for up to 72 hours and analyzed using ITLC. Rhenium-^188^-pyrophosphate (3.7 MBq in 100 μL) was added to 900 μL of freshly prepared human serum and stored at 37°C. At different time points (4, 24, and 48 hours after the reaction), 100 μL aliquots were removed and treated with 100 μL of ethanol (23). To precipitate the serum proteins, the samples were centrifuged at 3000 rpm for 10 minutes. Subsequently, chromatography of the supernatants was performed.

## 4. Results and Discussion

### 4.1. Preparation of Rhenium-188-Pyrophosphate

#### 4.1.1. Cold Phase Results

**Figure 1.**
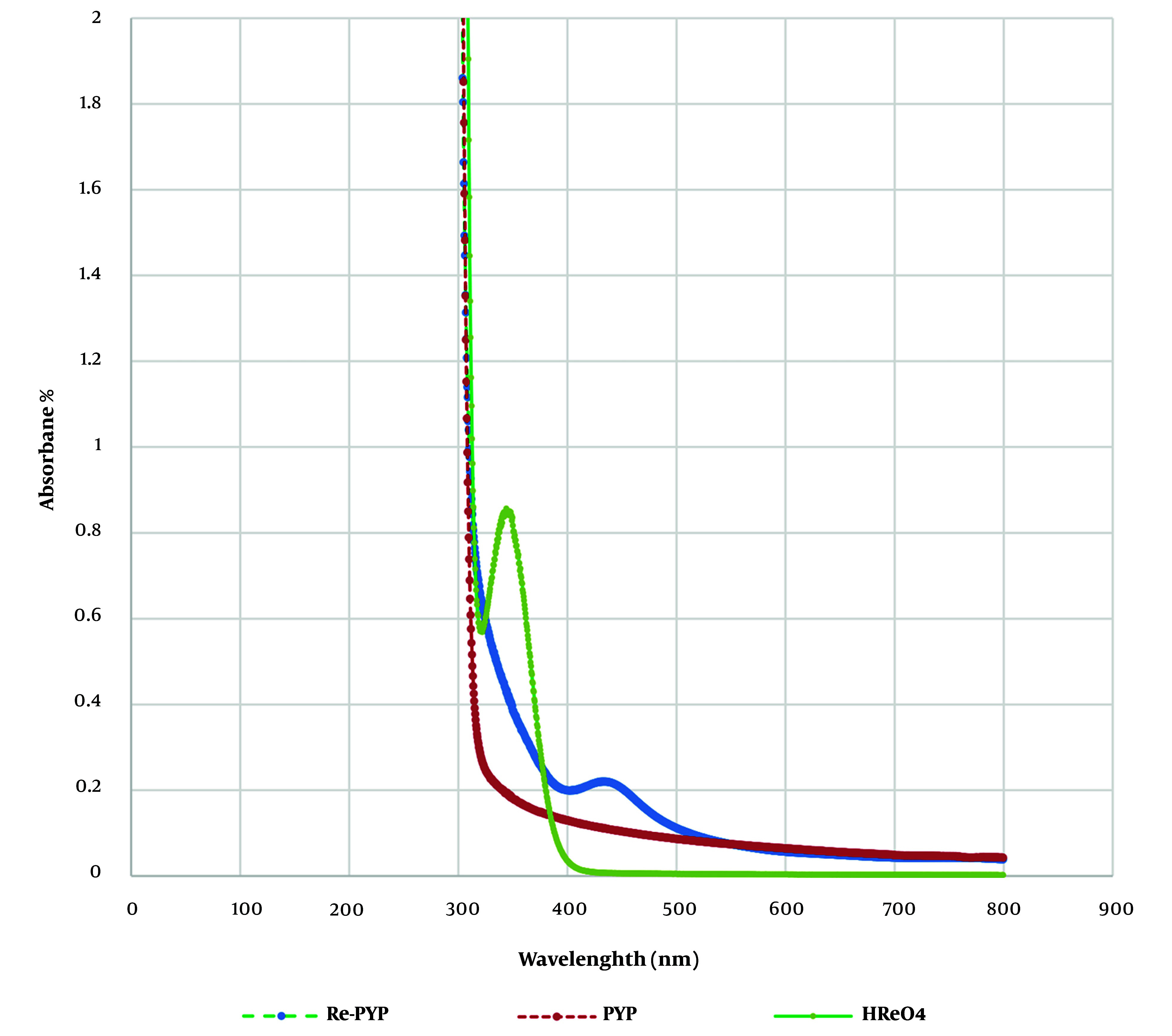
The UV-VIS spectrum of rhenium-pyrophosphate (PYP), PYP and HReO_4_

The UV-VIS spectrum is presented in Figure 1. Figure 1 shows the absorption spectrum of natural ReO_4_^−^ in the presence and absence of PYP. In the spectrum of aqueous natural ReO_4_^−^, absorption was evident at 350 nm, showing a strong band, which is consistent with other investigations (24). However, in the presence of PYP, the λmax shifted to 450 nm. This phenomenon is related to the ligand-to-metal charge transfer (LMCT) from the ligand to the solvent in the complex, which stabilizes the bond between ReO_4_^−^ and PYP. As ^99m^Tc-PYP is widely used in clinical applications and the chemical properties of rhenium are similar to technetium due to their placement in the 7th group of the periodic table, it can be assumed that the binding between rhenium and PYP could be similar to the binding of technetium and PYP. Rhenium typically has oxidation states of +5, +3, or +1, although it also exists in an oxidation state of +7 in ^188^ReO_4_, produced in the reactor.

**Figure 2.**
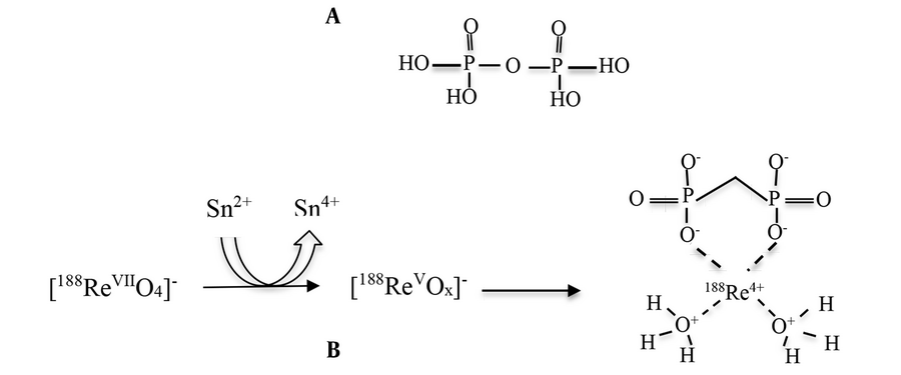
A, Pyrophosphate (PYP) structure, B, the possible formation and structure of rhenium-^188^-pyrophosphate (^188^Re-PYP)

The good affinity of rhenium for nitrogen, oxygen, phosphorus, and sulfur in oxidizing conditions makes it possible to label ligands with rhenium (24). The procurement of ^188^Re radiopharmaceuticals is straightforward: a kit containing a reducing agent, often stannous chloride, and a weak chelating agent is treated with the generator eluate (ReO_4_^−^). The compound is then incubated for a short time, usually at room temperature, resulting in a pure therapeutic agent ready for injection. The possible formation and structure of ^188^Re-PYP are shown in Figure 2. 

#### 4.1.2. Hot Phase Results

Various amounts of PYP ligand, bulk SnCl_2_, and pH were tested to study the effect of these parameters on the reaction performance of the compound complexation and purity. In the first stage, natural rhenium, as HReO_4_, was applied. The amount of ligand varied between 4 and 20 mg for the 0.08 mg amount of rhenium as HReO_4_ solution. A 98.96% ± 0.1% complex yield was obtained using 10 mg of PYP, 1 mg of SnCl_2_.2H_2_O, 0.3 mg of ascorbic acid, and 0.08 mg of potassium perrhenate as a carrier in 1 mL with 370 MBq of ^188^ReO_4_^−^ at pH 5 after 30 minutes of incubation at room temperature, leading to a specific activity of 30.22 mCi/mg.

**Figure 3.**
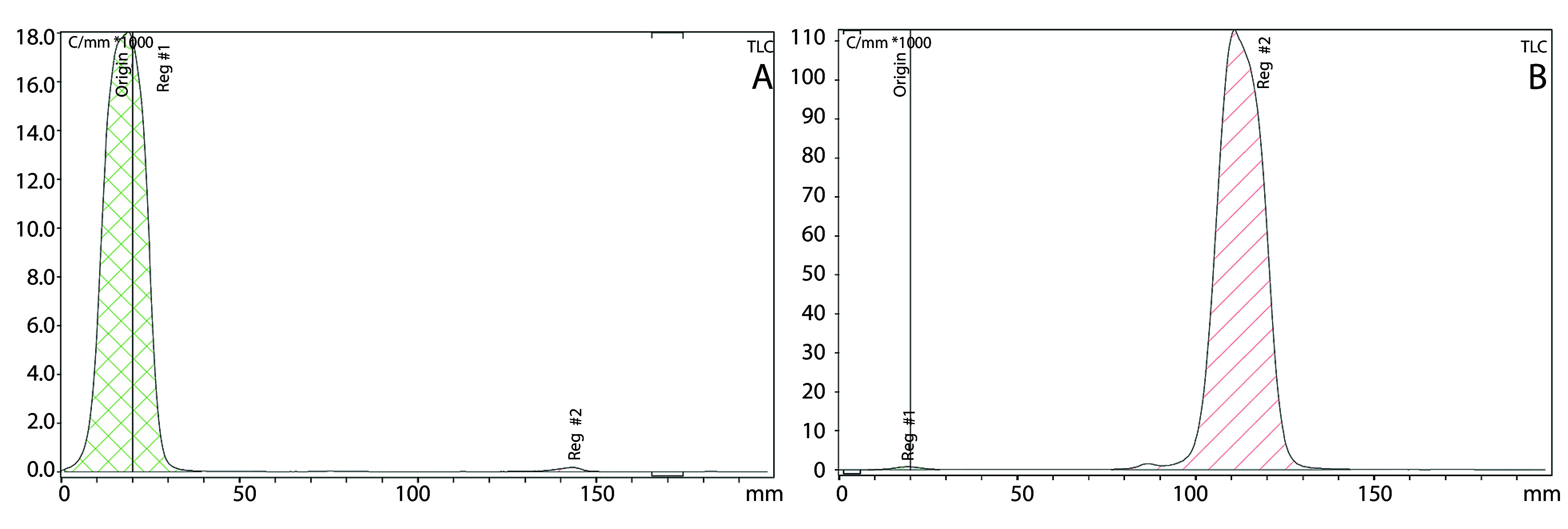
A, ITLC of rhenium-^188^-pyrophosphate (^188^Re-PYP) complex by acetone as mobile phase. B, ITLC of ^188^Re-PYP complex by saline (%0.9) as mobile phase

In the hot phase, a stability test was performed using two ITLC systems. For the first system, acetone was chosen as the mobile phase, where the Rf value for ReO_4_^−^ ion was one, and for the compound with ReO_2_, it was zero (Figure 3A). For the second system, saline (0.9%) was the mobile phase, where the Rf value for ReO_4_^−^ ion and the compound was one, and for ReO_2_, it was zero (Figure 3B). These two ITLC systems support the formation of the compound.

**Figure 4.**
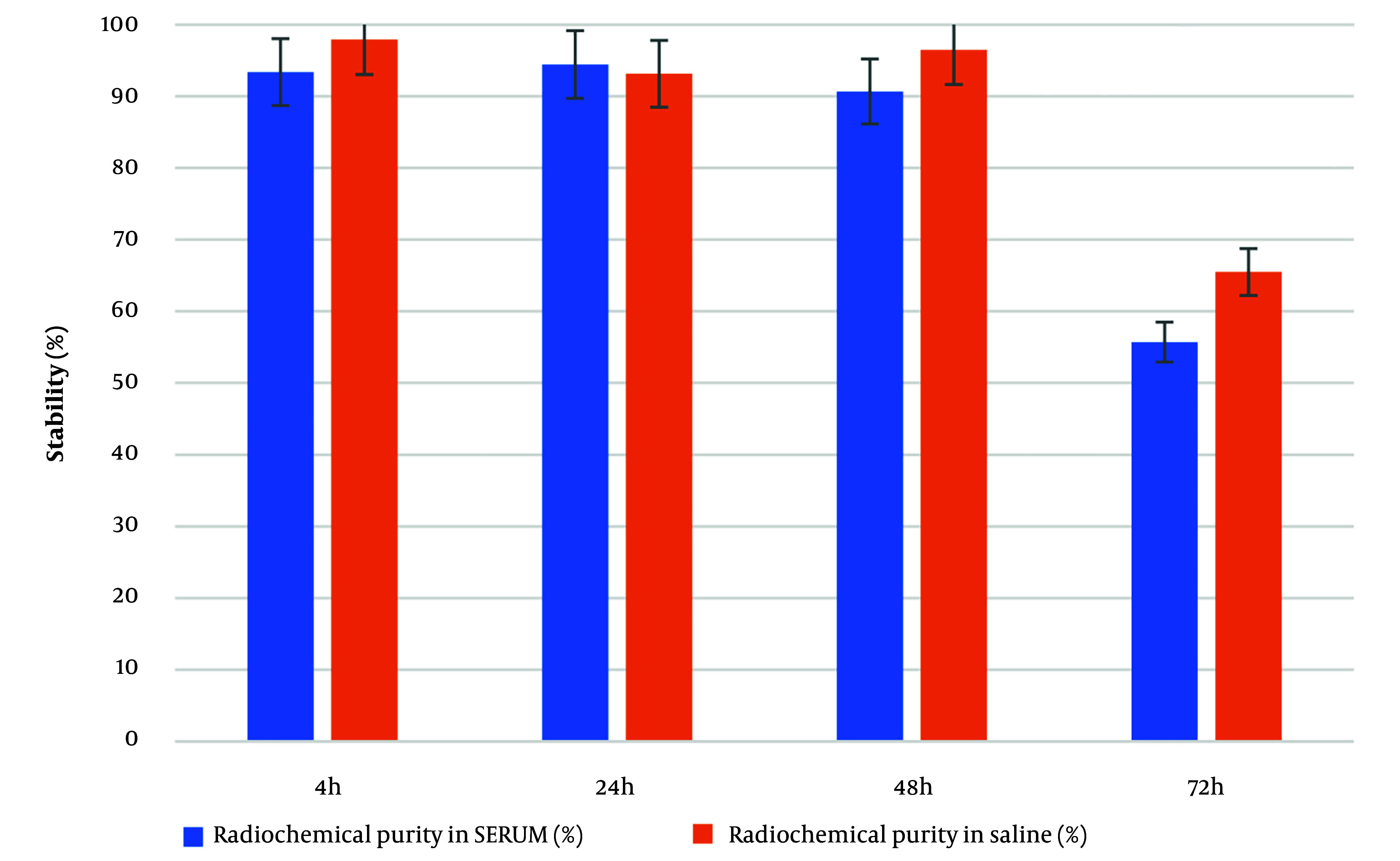
Radiochemical purity in human serum and in saline

The radiochemical purity and complexation yield of the compound in human serum at 37°C and in saline over 72 hours are shown in Figure 4. The compound's stability in human serum was 90.86% up to 48 hours after complexation. This stability result up to 48 hours is comparable to the half-life of ^188^Re; therefore, it can be concluded that ^188^Re-PYP is stable during its period of efficacy.

### 4.2. Biodistribution Investigation

**Figure 5.**
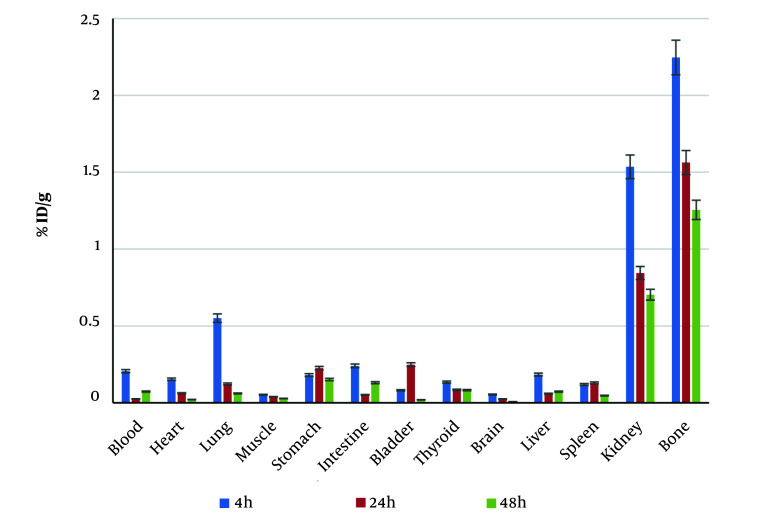
Biodistribution of rhenium-^188^-pyrophosphate (^188^Re-PYP) in mice

To evaluate the biodistribution of the radiolabeled ligand, BALB/c mice were utilized. The results, shown in Figure 5, confirmed that ^188^Re-PYP had maximum affinity for bone after 4 hours, with an uptake of 2.24% ± 0.667% ID/g. The maximum uptake for the kidney, spleen, and liver was 1.53% ± 0.378%, 0.13% ± 0.086%, and 0.18% ± 0.12% ID/g, respectively. The second highest uptake was in the kidneys, indicating that the radiopharmaceutical was eliminated through the renal system, while accumulation in vital organs such as the spleen and liver was negligible. These results reflect that the uptake of ^188^Re-PYP is primarily by bone and it is excreted by the renal system.

For comparison with other bone-seeker radiopharmaceuticals, the specific activities of ^153^Sm-EDTMP (7), ^186^Re-HEDP (6), and ^90^SrCl2 (16) were reported as 20, 35, and 40 mCi/g, respectively, while the specific activity of ^188^Re-PYP in this work was 30.22 mCi/g. This result shows that the specific activity of ^188^Re-PYP is comparable to clinical bone-seeker radiopharmaceuticals. The advantages of ^188^Re-PYP include easier labeling, with a maximum yield (> 99%) achieved by incubation for only a few minutes at room temperature, whereas other radiopharmaceuticals require at least 30 minutes of incubation at 100°C. Another advantage of ^188^Re-PYP is its in vitro stability, which is beneficial for clinical applications.

The difference in organ activity uptake between ^18^8Re-PYP and ^99m^Tc-PYP could be due to the application of a microfilter to remove any particles at the microscale, which reduced the activity of the solution by a factor of ten. The lower uptake in the liver might result from this filtration. Although the PYP kit used in this investigation is the clinical kit for ^99m^Tc, in clinical evaluation, the ^99m^Tc-PYP uptake of the liver is low enough to enable scanning of cardiac amyloidosis. Furthermore, an investigation by Jankovic et al. (25) showed that varying the molarity of PYP resulted in different liver uptake by a factor of 20, which was lower than the uptake in bone, similar to this investigation.

### 4.3. Conclusions

The present study investigated the initial labeling efficiency of ^188^Re-PYP, along with its biodistribution and in vitro stability. The ^188^Re-PYP conjugate was prepared under optimized conditions (10 mg of PYP, 1 mg of SnCl_2_.2H_2_O, 0.3 mg of ascorbic acid, and 0.08 mg of potassium perrhenate as a carrier in 1 mL with 370 MBq of ^188^ReO_4_^−^ at pH 5 after 30 minutes of incubation at room temperature), revealing radiochemical purity and stability. The biodistribution of the compound in mice exhibited high affinity for bone, while the complex was eliminated through the kidneys.

To compare ^188^Re-PYP with ^99m^Tc-PYP, it was noted that labeling with ^188^Re is more challenging than with ^99m^Tc. In this research, it was necessary to increase the ligand-to-metal ratio to achieve maximum radiochemical purity. Labeling PYP with ^188^Re represents a significant advancement in the field of radiopharmaceuticals, particularly for therapeutic applications to alleviate severe pain arising from tumor metastases in bone. The incorporation of ^188^Re, a beta-emitting radioisotope, into PYP molecules extends its potential from purely diagnostic applications to include therapeutic uses. However, to fully establish the benefits of this radiopharmaceutical for clinical application, further experiments in larger animal models are needed.

## Data Availability

The dataset presented in the study is available on request from the corresponding author during submission or after publication. The data are not publicly available due to privacy but they are available on request.
